# Effect of Polycarboxylate-Silane Modified Graphene Oxide Composite on the Properties of Cement Pastes

**DOI:** 10.3390/ma15155313

**Published:** 2022-08-02

**Authors:** Shuang Liu, Shiyu Li, Qin Wang, Ruifeng Zhang, Xiao Liu

**Affiliations:** 1 Beijing Building Materials Academy of Sciences Research, Beijing 100041, China; liushuang@bbma.com.cn; 2 State Key Laboratory of Solid Waste Reuse for Building Materials, Beijing 100041, China; 3 Beijing Key Laboratory of Functional Materials for Building Structure and Environment Remediation, Beijing University of Civil Engineering and Architecture, Beijing 100044, China; lishiyu@emails.bjut.edu.cn (S.L.); 201602060126@stu.bucea.edu.cn (R.Z.); 4 Key Laboratory of Advanced Functional Materials, Faculty of Materials and Manufacturing, Ministry of Education, Beijing University of Technology, Beijing 100124, China; liux@bjut.edu.cn

**Keywords:** modified, graphene oxide, mechanism, workability

## Abstract

As a nano-carbon material with excellent properties, Graphene oxide (GO) has been widely used in cement-based materials, and the negative effect of paste workability caused by GO agglomeration has also been widely concerning. In this study, a polycarboxylate-silane modified graphene oxide composite (PSG) was prepared by coupling polycarboxylate molecules to the surface of graphene oxide (GO) via a reaction with vinyl triethoxysilane. The effects of GO and PSG on the cement paste and the mechanisms underpinning these effects were investigated using fluidity and rheological parameter measurements, and ion concentration and zeta potential analyses. It was found that, in the aqueous phase of the paste, the polycarboxylate molecular chains on the surface of the PSG complexed with calcium ions (Ca^2+^), thereby preventing Ca^2+^ from bridging the GO sheets, and thus stabilizing the surface potential and the electrostatic repulsion. This prevented the PSG from forming an agglomerate structure such as that formed by GO under the same conditions, thereby substantially enhancing workability of paste with nano-carbon material. This study provides some new foundations and ideas for the further application of graphene oxide materials in cement-based materials.

## 1. Introduction

With new technologies, high-rise buildings are becoming increasingly structurally specialized and complex. This places greater demands on the performance of concrete as a structural material. As the main component of concrete, fresh cement paste plays a key role in the suspension lubrication of concrete before condensation hardening, so the workability of cement paste is the main guarantee of concrete homogeneity and pumpability [1]. As the flow and rheological properties of cement paste are a good indication of its homogeneity and pumping properties, the characterization of these two aspects is particularly important for both theory and application.

In recent years, it has been found that adding graphene oxide (GO) and other new nano-carbon materials to cementitious materials can improve the strength and toughness of the matrix. However, doping cement with GO substantially increases the viscosity of the cement paste, greatly reducing its fluidity and significantly changing its rheological properties [2]. This greatly limits the application of GO-doped cement-based materials. To improve this process, the mechanism of the influence of GO on the fluidity and rheological properties of cement paste must be determined.

Shang et al. [3] added silica fume (SF) and/or GO into cement and investigated the fluidity and rheological properties of the resulting pastes. Their results showed that either GO or SF alone could reduce the fluidity, yield stress, and other rheological parameters of the paste, and that doping with both GO and SF led to a morphological synergy effect, such that the fluidity and rheological properties of their paste were significantly better than those of GO- or SF-doped paste. Qin et al. [4] investigated the interaction between GO and fly ash in a cement-fly ash system and found that fly ash reduced the negative effect of GO on cement paste workability via a balling effect and decreased water demand, and the GO-mediated increased viscosity kept the fly ash in suspension and thereby prevented paste dilution.

In addition, other studies have shown that the modification of GO with superplasticizer, which is common in engineering, can partly offset the GO-mediated increase in the viscosity of cement paste. Lv et al. [5] mixed a naphthalene sulfonate agent with GO under ultrasonic conditions and combined the resulting mixture with cement paste. They found that the adsorption of the plasticizer molecules on the surface of the GO sheet improved its dispersion in the cement paste and thus decreased the viscosity of the resulting paste. Zongjin et al. [6] mixed polycarboxylate superplasticizer (PCE) and GO into a cement paste and observed that the GO was more stably dispersed in the resulting alkaline cement paste.

The above research shows that the negative effects of GO on cement can be improved by mixing GO with superplasticizers and adding the resulting mixture to cement paste. However, most of the modification methods involve physical adsorption or the formation of intercalated structures of superplasticizer molecules and GO. As a result, it is difficult to separate and purify the dopant mixtures before their addition to cement paste, which makes the combination of dopant mixtures with cement paste a complicated process. Consequently, there is a need for modified GO dopants with stable physicochemical properties that can be separately purified and easily added to cement pastes.

The surfaces of GO sheets are decorated with a large number of oxygen-containing functional groups, such as hydroxyl groups and carboxyl groups, which gives considerable scope for the modification of GO surface chemistry. Thus, in recent years, some researchers have grafted superplasticizers onto GO. Gao et al. [7] directly modified GO with water-reducing monomers such as polyoxyethylene ether methacrylate monomethyl ester and methacrylic acid using ammonium peroxide as an initiator. Li et al. [8] modified GO by ultrasonically mixing a polycarboxylate superplasticizer with GO at 60 °C. However, the free-radical polymerization mechanism of the reaction of a polycarboxylate superplasticizer and GO involves a reaction between alkene monomers, rather than a reaction with the oxygen-containing groups on the surface of the GO sheet. In addition, the double bonds visible in the infrared spectrum of GO are attributable to large off-domain π-bonds formed by the hybridization of the internal sp^2^ carbon atoms. These species have insufficient alkenyl character to participate in free radical polymerization reactions, so there is more to be discovered about the mechanism underlying this process.

To link GO and monomers of polycarboxylate via alkenyl groups, the GO needs to be alkenylated, i.e., a molecule having one or more free alkenyl molecule needs to be grafted onto its surface. Silane coupling agents are the most commonly used inorganic-organic interface bridging agents and have become the preferred GO modifiers. In related studies, many researchers have investigated the surface modification of GO using silane coupling agents. Shijingjing et al. [9] reacted γ-methacryloxypropyltrimethoxysilane with the -OH group on the surface of GO, then reduced the resulting product with hydrazine hydrate to obtain silane-functionalized graphene. This functionalized graphene had an increased degree of surface disorder and an increased degree of peeling in the solvent. Tian Chen et al. [10] used polymethyl methacrylate-*n*-propyltrimethoxysilane to modify the surface of GO, which resulted in improved dispersion in the silicone-packing matrix and thus afforded GO-silicone composites with higher thermal conductivity.

In this study, a pretreatment modification of the GO surface was carried out using a silane coupling agent containing a vinyl group, which was attached to the GO via a carbon-carbon double bond and polymerized with the relevant monomer to complete the grafting of polycarboxylate molecules onto the GO surface. An analysis of the effects of GO and PSG on the fluidity and rheological properties of cement paste was conducted, which led to explanations of the underlying mechanisms.

## 2. Experiment

### 2.1. Materials

**Figure 1:**
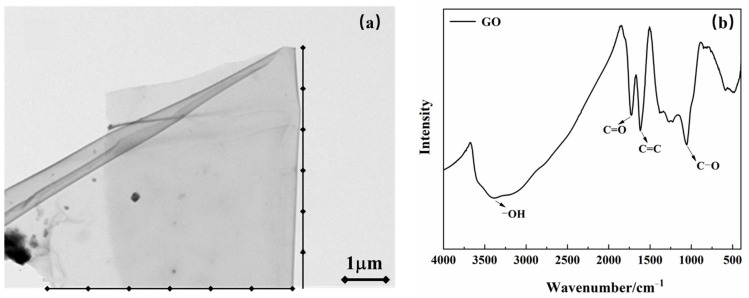
Microscopic morphology and functional-group composition of GO: (**a**) transmission electron microscopy image; and (**b**) Fourier-transform infrared spectrum.

Reference cement produced by Shandong Lucheng Cement Co., Ltd. was used, and its chemical composition is shown in Table 1. The GO powder is a high-activity GO powder produced by Shanxi Institute of Coal Chemistry, Chinese Academy of Sciences. Prepared by the Hummers method, it is a fine brownish powder with a purity of ≥98.5%; the microstructure and chemical group composition are shown in Figure 1a,b. The GO powder has a flaky structure and contains oxygenated groups such as carboxyl groups and hydroxyl groups.

The silane coupling agent was vinyl triethoxysilane (VTEO) produced by Nanjing Chuangshi Chemical Auxiliary Co., Ltd. This compound contains one reactive vinyl and three reactive ethoxy groups and can be hydrolyzed under acidic conditions.

**Figure 2:**
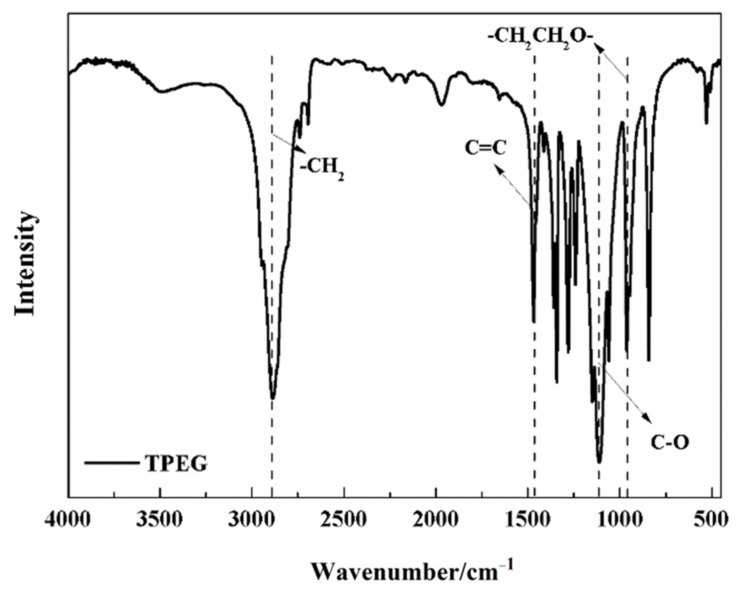
Fourier-transform infrared spectrum of TPEG.

The polyether macromonomer used for compound synthesis and polycarboxylate superplasticizer synthesis was methallyl poly(ethylene glycol) ether (TPEG), produced by Liaoning Aoke Chemical Co., Ltd. The relevant physicochemical indicators and infrared spectra of TPEG are shown in Table 2 and Figure 2, respectively, from which the -CH_2_ group, C=C group, C-O group, and glycol structure group can be identified.

### 2.2. Synthesis of Polycarboxylate-Silane Modified Graphene Oxide (PSG) Composite

A quantity of 15 mL of 95% aqueous ethanol and 0.3 g GO were combined in a conical bottle, and the resulting mixture was shaken well and then placed in an ultrasonic disperser for 4 h (ultrasonic power 270 W, frequency 35 kHz, temperature less than 30 °C). Then, 1 g of VTEO was added, and the resulting mixture was shaken well and placed in an ultrasonic disperser for approximately 2 h at a control temperature of 30–40 °C. Next, the mixture was stirred for approximately 3 h in a water bath at 80 °C. Finally, the solids were subjected to vacuum filtration and vacuum drying to yield the silane-modified graphene oxide (SGO) product.

TPEG and a certain volume of deionized water were placed in four flasks. A quantity of 30% hydrogen peroxide solution was added to each mixture as an initiator, and the flasks were placed in a 40 °C water bath to ensure complete dissolution of the TPEG.

A certain amount of SGO, 4.7 g of acrylic acid, and a certain volume of deionized water were combined in a conical flask, and then placed in an ultrasonic disperser for approximately 1 h (ultrasonic power 270 W, frequency 35 kHz, temperature less than 30 °C). The resulting mixture was labeled “Solution A.” Then, 0.20 g mercaptoacetic acid, 0.1 g ascorbic acid, and a certain volume of deionized water were combined and mixed well in a conical bottle, and the resulting mixture was labeled “Solution B.” 

Next, PSG was formed by concurrent dropwise addition of Solution A (over 2 h) and Solution B (over approximately 2.5 h) to a flask in a 40 °C water bath. After the dropwise addition of both reactants was complete, the reaction mixture was stirred at 40 °C for 1 h. After the reaction was complete, the product was obtained by filtration, washed repeatedly with 20 mL deionized water, and dried at 80 °C for 24h. The dried PSG product was then carefully grinding with an agate mortar until no coarse particles were visible and stored in a drying dish.

### 2.3. Methods

#### 2.3.1. Characterization of Materials

Fourier-transform infrared (FTIR) spectroscopy was used to distinguish the different functional groups and morphologies in the products. The samples to be tested were sufficiently ground with sodium bromide crystals to give a homogeneous mixed powder, which was then pressed into light-translucent circular sheets in a tablet press. These sheets were placed in the FTIR instrument for analysis. X-ray diffraction (XRD) analysis was used to study the differences in the crystal structures of the synthetic products and raw materials. This was performed by placing a sufficiently dry sample in the test mold and mounting this in the instrument. The scan range was 5–90°, the step length was 0.02°, and the scan-rate was 6°/min, with a copper target being used. Thermogravimetric-differential thermal analysis (TG-DTA) was used to examine the thermal and content changes of the surface groups of GO and SGO. Briefly, samples were dried well and then placed in an alumina crucible in the instrument, together with the reference sample, and heated at 10 °C/min under a nitrogen atmosphere.

#### 2.3.2. Fluidity Test

A product fluidity test was performed according to Chinese national standard GB/T8077–2000 “Methods for testing uniformity of concrete admixture.” The water–binder ratio was 0.3, and the specific mix ratio is shown in Table 3. The height of the mold used was 60 mm, the upper diameter was 36 mm, and the lower diameter was 60 mm. Timing began when the mixing was completed, and the diameter of the spreading paste was measured at 0 min, 30 min, 60 min, 90 min, and 120 min. At each of these time-points, the vertical diameter was measured twice, and the average was recorded as the final test result. Deionized water was used to give both dispersions (GO and PSG) a concentration of 2 mg/mL. The proportion of synthetic raw materials in PCE solution was 15%, which was the same as the proportion of PSG. As the density of the GO and PSG dispersions was the same as water (1 g/cm^3^) and the proportion of materials present in the dispersions was extremely low, the dispersion was treated as a direct replacement for part of the mixing water. The overall test temperature was controlled at 25 ± 3 °C.

#### 2.3.3. Rheological Measurements

The mix ratio in Table 3 was also used for the rheology test of cement paste. The test pastes were prepared as follows. When mixing the paste, first place the powder in a plastic beaker, then mix the go or PSG dispersion, water reducer and mixing water, and finally add the mixed solution to the powder. Then, the remaining test powder was added, and the resulting dispersion was mixed at 62 r/min for 90 s, after which the dispersion was visually inspected for 15 s to confirm its uniformity. This being confirmed, mixing was continued at 140 r/min for another 90 s. Next, the resulting paste was quickly loaded (within 30 s) into the measuring drum of the rheometer, and the loaded drum was placed on the rheometer to be measured. Measurement was performed according to the setting procedure, with the change of shear rate set to a lift cycle with 15 shear-rate points, making the initial and final rate of the lift stage 5 s^−1^ and 250 s^−1^, respectively [11]. To investigate the changes in the yield stress, plastic viscosity, and shear deformation of the cement pastes of different groups, the Modify Bingham model (M–B model) and the Herschel–Bulkley model (H–B model) were used to analyze the rheological data of the pastes. 

The M–B model and its basic equivalent, the Bingham model, are both commonly used for fitting analysis models in cement rheology. The mathematical expressions of both models contain two parameters, yield stress (τ_0_) and plastic viscosity (τ_0_). The M–B model considers the pseudoplasticity of the paste on the basis of the Bingham model, and the corresponding mathematical corrections are made to the basic model to bring the fitting results closer to the actual situation [12]. Therefore, the M–B model was selected for analytical fitting in the analysis of yield stress and plastic viscosity. The mathematical expression of the M–B model is shown in Equation (1).

τ = τ_0_ + η_p_ γ + cγ^2^ (1)

When the M–B model was used to analyze the paste, the shear deformation of the paste was not well characterized [13], so the H–B model was used instead, as the latter model can better characterize the pseudoplastic plasma. The mathematical expression of the H–B model is shown in Equation (2), as follows:

τ = τ_0_ + kγ^n^ (2)

τ and τ_0_ represent the test shear-stress and slurry yield-stress (in Pa); γ is the test shear-rate (in s^−1^); η_p_ represents the slurry plastic viscosity (in Pa·s); and n and k represent the paste pseudoplasticity coefficient and consistency coefficient, respectively, which are both dimensionless parameters.

#### 2.3.4. Inductively Coupled Plasma-Optical Emission Spectroscopy (ICP-OES) Analysis

To study the interaction of GO and PSG with the most prevalent ions in the aqueous phase of the cement paste, the effects of GO and PSG dispersions on Ca^2+^ concentration and pH in solution were investigated using inductively coupled plasma emission spectroscopy (ICP-OES) and pH meters in a saturated calcium hydroxide (Ca(OH)_2_ solution (CHS), calcium nitrate (Ca(NO_3_)_2_) solution (CAS), and sodium hydroxide (NaOH) solution (NHS). The dosages of GO and PSG were both 0.03%, and the amount of the required dispersion and corresponding solution was calculated based on a 0.35 water-to-binder ratio. The mixing ratio is shown in Table 4. The samples were placed in a shaker for approximately 30 min at 25 °C, and the pH of each sample was then tested with a pH meter. Each sample was centrifuged at 13,500 r/min for 10 min, and the resulting supernatant was passed through a 0.22-μm needle filter to obtain the ion-concentration test specimen.

#### 2.3.5. Zeta Potential Test

GO and PSG form homogeneously dispersed colloids in aqueous dispersions and measuring the surface zeta potential of particles gives a good indication of their surface electrical properties and stability in different systems. This was performed with a JS94H microelectrophoresis instrument produced by Shanghai Zhongchen Digital Technology Equipment Co., Ltd. Each sample was loaded into a quartz cuvette, and the cuvette was placed in the sample tank and analyzed according to the standard procedure.

#### 2.3.6. Dispersion Characterization

The mesoscopic state of GO and PSG in aqueous phase systems of different composition was observed on an ultra-depth microscope (VHX-6000, Keynes, Japan). The test samples had the same composition as those in Table 4, and each sample was placed via dropper onto a glass slide, covered with a coverslip, and then viewed at 500× and 1000× magnification.

## 3. Results and Discussion

### 3.1. Product Characterization

**Figure 3:**
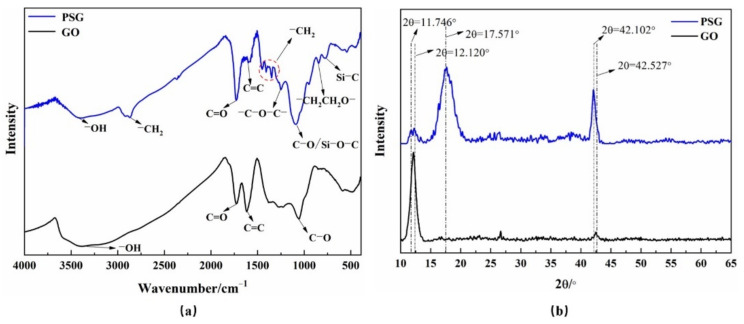
FTIR and XRD spectra of GO and PSG: (**a**) FTIR; and (**b**) XRD.

The FTIR spectra of GO and PSG are presented in Figure 3a. It can be seen that, due to the introduction of the polycarboxylate molecular chain, the PSG, while retaining some of the characteristic groups of GO, obtained new absorption peaks at 2877 cm^−1^, 1355 cm^−1^, and 833 cm^−1^, corresponding to methylene groups (−CH_2_), ether bonds (−CH_2_CH_2_O−), and glycol groups (−CH_2_CH_2_O−), respectively [14], which were not present in the GO. In addition, the absorption peak intensity of the double bond in the PSG was significantly less than that in the GO, indicating that much of the double-bond character of GO was consumed during its conversion to PSG.

The XRD results of GO and PSG are presented in Figure 3b. The PSG retained two characteristic peaks, 2 θ = 11.746° and 2 θ = 42.102°, but a new, smoother peak also appeared at 2θ = 17.571°, caused by the introduction of polycarboxylate molecules. Specifically, during the drying process, the polycarboxylate polymer chains on the surface of the PSG samples were curled and cross-linked, thus forming a new layer of polymer chains on the surface. This layer had a particular crystal structure and therefore a new diffraction peak appears in the map [15].

**Figure 4:**
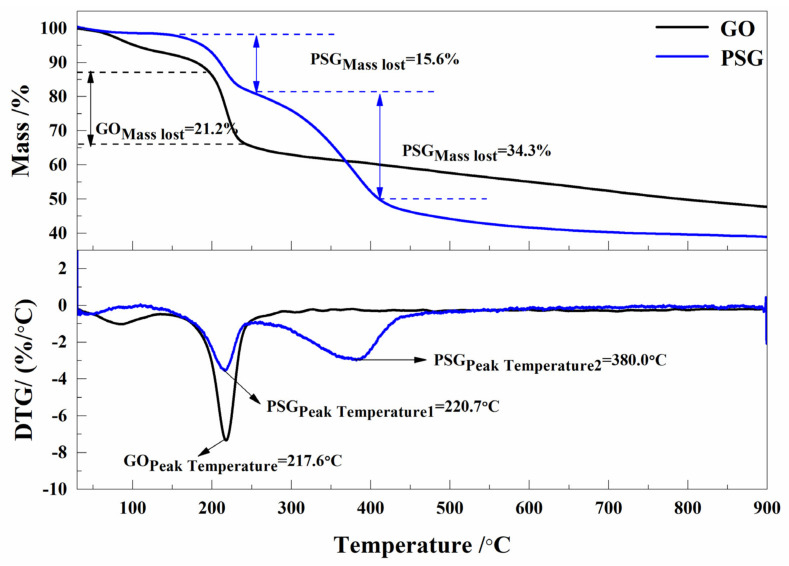
TG-DTA curve of GO and PSG.

Figure 4 shows the results of the TG-DTA of GO and PSG. It can be seen that the first weightless stage in the TG-DTA curve of PSG was consistent with the TG-DTA curve of GO. However, there was a second phase of weightlessness in the TG-DTA curve of PSG, corresponding to the decomposition of the polyoxyethylene ether component of the polycarboxylate molecule, clearly indicating the grafting of the polycarboxylate onto the surface of the GO sheet.

**Figure 5:**
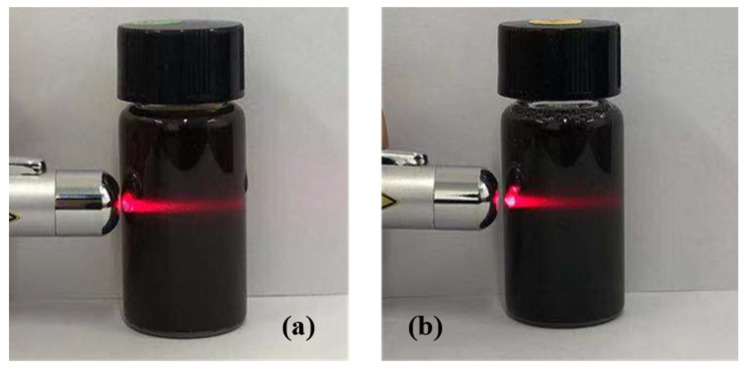
Characterization of aqueous dispersion of GO and PSG: (**a**) GO; and (**b**) PSG.

The dispersion states of the GO and PSG aqueous dispersions are shown in Figure 5a,b. As can be seen, both were black translucent homogeneous dispersions, and a clear light pathway appeared inside the dispersion under red-laser irradiation, confirming the existence of a Tindal effect [16,17]. This result indicated that both GO and PSG can form a well-dispersed colloidal system in an aqueous phase system.

**Figure 6:**
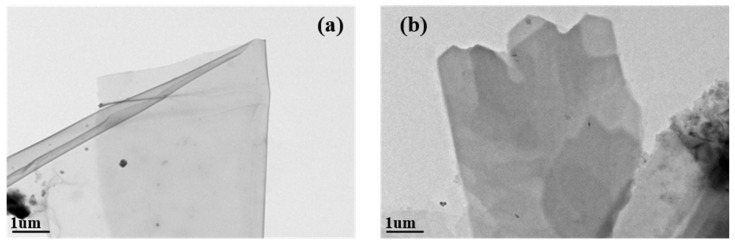
TEM of GO and PSG: (**a**) GO; and (**b**) PSG.

The TEM images of GO and PSG are presented in Figure 6a,b. It can be seen that the GO (Figure 6a) was a transparent thin-gauze-like sheet; the PSG (Figure 6b), although it retained the sheet structure of GO, and had a translucent/waxy surface layer, unlike the transparent GO layer. This is due to the grafted polycarboxylate molecules forming a polymeric layer on the surface of the sheet. In addition, there were structural defects visible at the edge of the layer, which were the result of its modified structure. Thus, these TEM images confirmed the results of the XRD analysis and TG-DTA.

### 3.2. Effect of GO and PSG on the Fluidity of Cement Pastes

**Figure 7:**
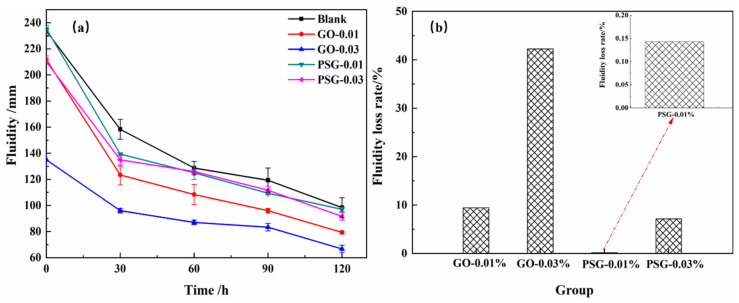
Results of cement paste fluidity test: (**a**) time dependency of fluidity; and (**b**) initial fluidity loss-rate of cement paste.

Figure 7a shows the results of the fluidity tests of GO- and PSG-doped cement paste over time. It can be seen that the initial fluidity of the slurry doped with GO was significantly lower than that of the blank sample, whereas the fluidity of the slurry doped with PSG was not significantly lower. Over time, the fluidity of each group gradually decreased, and the fluidity of the slurry with PSG became similar to that of the blank, whereas the fluidity of the slurry with GO was significantly lower than that of the blank.

Figure 7b is a comparison of the initial fluidity-loss rate of the cement paste. Compared with the blank, the initial fluidity of the cement slurry with 0.01 wt% and 0.03 wt% GO decreased by 9.42% and 42.2%, respectively. The fluidity of the sample with a PSG content of 0.01 wt% was similar to that of the control, having a loss rate of only 0.15%; when the PSG content was 0.03 wt%, the loss rate was much less than the corresponding 0.03 wt% GO rate of 42.2%. These data showed that PSG decreased the fluidity of cement paste substantially less than GO did.

### 3.3. Effect of GO and PSG on the Rheological Properties of Cement Pastes

#### 3.3.1. Effect of PSG on the Plastic Viscosity and Yield-Stress of Cement Pastes

**Figure 8:**
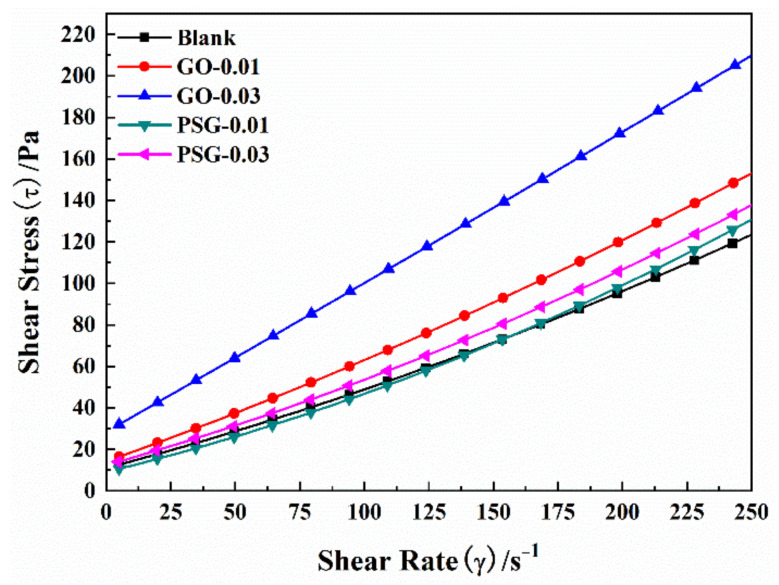
Diagram of M–B model-fitting result.

Figure 8 and Table 5 show the curves and results obtained by fitting and analyzing the rheological data using the M-B model with the objective function (1). The model was used to analyze the yield stress (τ_0_) and plastic viscosity (η_p_) of the slurry.

It can be seen from Table 5 that the yield stress and plastic viscosity of the cement paste slurry doped with GO increased as the GO content increased. Thus, the yield stress of the slurry was 14.22 Pa and 27.84 Pa when the GO content was 0.01 wt% and 0.03 wt%, respectively, which was 29.17% and 152.88% greater, respectively, than the yield stress of the blank (11.01 Pa). The plastic viscosities of 0.01 wt% and 0.03 wt% GO-doped cement paste were 0.44 Pa·s and 0.75 Pa·s, respectively, which was 34.14% and 126.89%, greater, respectively, than the plastic viscosity of the blank (0.33 Pa·s). 

In contrast, the yield stress and plastic viscosity of the cement paste doped with PSG did not change greatly with the increase in PSG content. Compared with the blank, the yield stress of the slurry with PSG content of 0.01 wt% and 0.03 wt% increased by 10.40% and 21.79%, respectively. However, the plastic viscosity changed by −8.46% and 6.34%, respectively, compared to the blank. From these results, it can be seen that the incorporation of PSG did not significantly affect the yield stress or plastic viscosity of the resulting cement paste compared to the cement paste incorporating GO.

#### 3.3.2. Effect of PSG on Shear Deformation of Cement Pastes

**Figure 9:**
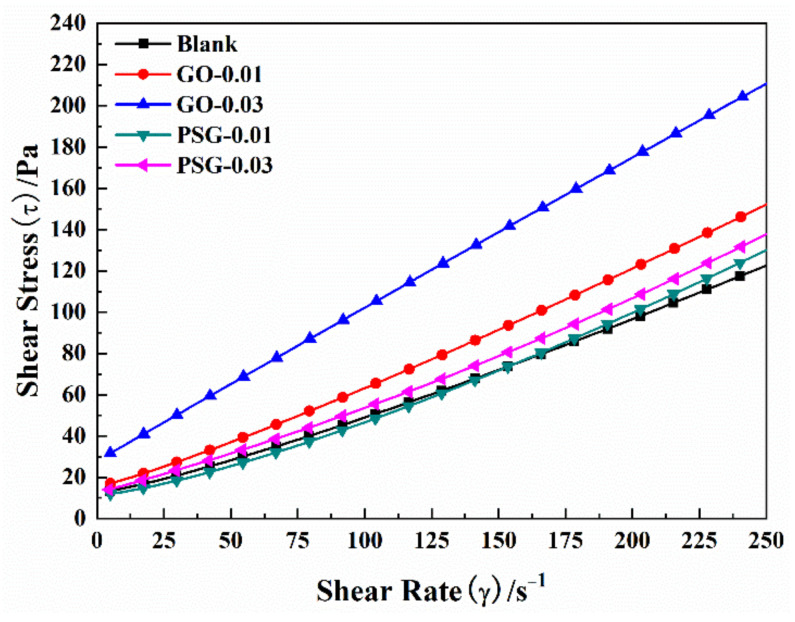
Diagram of H–B model-fitting result.

Figure 9 and Table 6 show the curves and related results obtained by fitting and analyzing the rheological data using the H–B model and the objective function (2). The model was used to study the effect of GO and PSG on the shear deformation of the slurry. The key parameter in this model was the pseudoplastic coefficient (n): when n > 1, the slurry displayed shear-thickening behavior, which increased with n. According to the fitting results, the yield stress results were in keeping with the fitting results of the M–B model.

It can be seen from Table 6 that the pseudoplasticity of the cement paste containing GO gradually decreased with the increase in GO content. When the GO content was 0.01% and 0.03%, the n-value decreased by 4.57% and 16.28%, respectively, with respect to the blank. The pseudoplasticities of the samples doped with 0.01% and 0.03% PSG were close to that of the blank, i.e., 1.50% and 5.23% more, respectively, than the blank. The results showed that the incorporation of GO weakened the shear thickening of cement paste, which affected the actions of the water reducers and cement particles. However, the incorporation of PSG did not significantly affect the shear-thickening of cement paste slurry within the test blending range, as the shear-thickening in the PSG-doped slurries was very similar to that of the blank.

### 3.4. Mechanism of the Effect of GO and PSG on the Properties of Cement Pastes

It can be seen from the results in Section 3.2 and Section 3.3 that the incorporation of GO or PSG into cement slurries yielded modified slurries with significantly different workability and rheological properties. Thus, the incorporation of GO had an obvious viscosity-increasing effect, and the desired performance of the slurry decreased sharply with the increase in GO. In contrast, the rheological properties and workability of the PSG-doped slurry were largely consistent with those of the blank group. 

Shang et al. [3] proposed that the viscosity-increasing effect of GO on cement slurry is due to the large, agglomerated structures formed by GO in slurries. These agglomerates envelop a portion of the free water in the slurry, thus reducing the amount of free mixing water available, and consequently reducing the fluidity of the slurry. Our previous research [4,11] confirmed that this was the case, and we also found that the large specific surface area of GO enabled it to adsorb some of the superplasticizer molecules, such that the effect of the superplasticizer was diminished, and the GO formed a recombinant and robust agglomerate with cement particles, thereby affecting the workability and rheological properties of the modified slurry. However, the incorporation of PSG did not have a significant effect on the workability and rheology of the cement slurry, indicating that PSG did not exhibit the same agglomerating tendency as GO.

#### 3.4.1. Comparative Studies of the Dispersion of GO and PSG in Different Solution Systems

**Figure 10:**
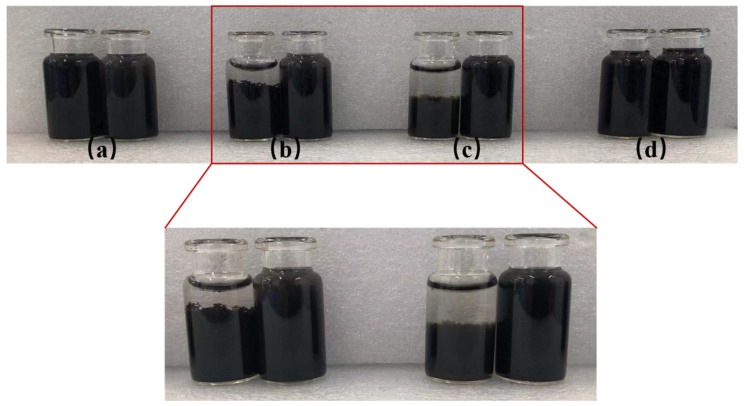
Dispersion effects of different systems: (**a**) [GO-W/PSG-W]; (**b**) [GO-CHS/PSG-CHS]; and (**c**) [GO-CAS/PSG-CAS]: (**d**) [GO-NHS/PSG-NHS].

Figure 10a–d shows the effect of GO dispersion and PSG dispersion in different systems, namely (water (W), Ca(OH)_2_ solution (CHS), Ca(NO_3_)_2_ solution (CAS), and NaOH solution (NHS). The results showed that GO-W and PSG-W mixed with distilled water did not exhibit significantly different appearances: both remained black translucent homogeneous dispersions (Figure 10a). In the samples mixed with the Ca(OH)_2_ solution, GO-CHS showed obvious agglomeration, and GO quickly separated from the water and settled to the bottom of the sample bottle. However, there was no obvious agglomeration in PSG-CHS, which remained a black translucent homogeneous dispersion similar to PSG-W (Figure 10b). PSG-CAS also showed no visible agglomeration when dosed with an equivalent Ca^2+^ concentration of CAS solution (Figure 10c). However, agglomeration was still observed in GO-CAS, although the agglomerates visually appeared to be less dense than those in GO-CHS. In the samples doped with NHS solution, no macroscopic segregation was observed in either GO-NHS or PSG-NHS (Figure 10d).

**Figure 11:**
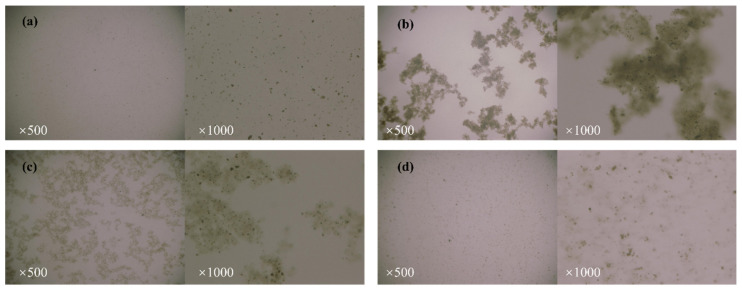
Ultra-depth field microscopic image of GO in different systems: (**a**) GO-W; (**b**) GO-CHS; (**c**) GO-CAS; and (**d**) GO-NHS.

**Figure 12:**
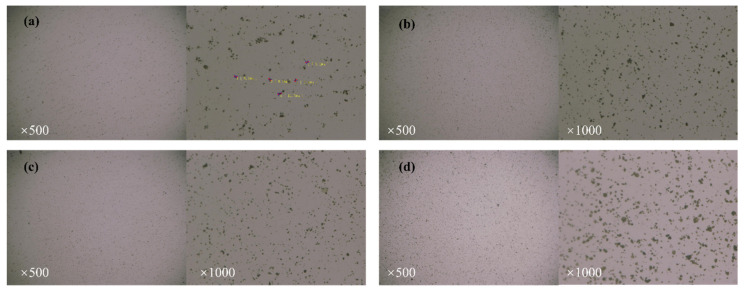
Ultra-depth field microscopy images of PSG in different systems: (**a**) PSG-W; (**b**) PSG-CHS; (**c**) PSG-CAS; and (**d**) PSG-NHS.

To further understand the morphological changes of GO and PSG in different systems, ultra-depth microscopy was used to observe their morphology. Figure 11a–d and Figure 12a–d are ultra-depth microscopic images of GO and PSG in different systems, respectively. It can be seen from Figure 11a–d that the particle morphology of GO varied in different solvents, i.e., distilled water, Ca(OH)_2_ solution, calcium nitrate solution, and sodium hydroxide solution. For example, Figure 11a, shows GO suspended in distilled water, and it can be seen that the black slice layer was difficult to recognize at 500× magnification, but could be more clearly recognized at 1000× magnification.

When GO was mixed with CHS solution, as shown in Figure 4, Figure 5 and Figure 6b, large, dense GO agglomerates were formed in the aqueous phase at 500× magnification. The pronounced internal structure of these agglomerates can be observed at 1000× magnification, notably showing many dense black punctiform particles (an observation also mentioned by Chuah et al. [18]). However, when GO was mixed in a CAS solution of an equivalent Ca^2+^ concentration, the resulting agglomerates (Figure 12c) were slightly smaller and less dense than those in the CHS solution, and although the same punctiform particles were visible, their number was significantly reduced. When GO was mixed with an NHS solution of equal OH^−^ concentration, as shown in Figure 11d, its state was similar to that in the distilled water sample at 500× magnification, whereas at a 1000× magnification there were some relatively diffuse, amorphous agglomerates.

Comparing Figure 12a,b, we can see that there was minimal overall variation in the appearance of PSG in the four systems. Thus, when PSG was mixed with distilled water (Figure 12a), the resulting mixture was substantially similar to that of the GO–distilled water mixture in Figure 11a, although some large agglomerates were visible at 1000× magnification. When PSG was mixed in a CHS solution and a CAS solution of an equivalent Ca^2+^ ion concentration, the agglomerates that were seen in the analogously doped GO mixture were not observed (compare Figure 12b,c with Figure 11b,c): in fact, the dispersions were largely the same in appearance as in Figure 12a. When PSG was mixed with an NHS solution of an equal OH^−^ concentration, no significant agglomeration was observed at either magnification. Overall, these data showed that PSG did not react with Ca^2+^ or OH^−^, either alone or together, to produce aggregates in the aqueous phase.

#### 3.4.2. Comparison of Zeta Potential between GO and PSG in Cement Solution

From the above results, it can be seen that GO exhibited distinctly different effects when interacting with the four solvent systems, whereas PSG did not (at the macroscopic to mesoscopic scale). However, both GO and PSG formed a homogeneous colloidal solution once in aqueous dispersion, so the surface of the particles in these dispersions should exhibit electrical properties. Specifically, the interaction of these different particles with various systems and electrolytes caused changes in the surface electrical properties of the particles, resulting in a change in the state of the colloids.

**Figure 13:**
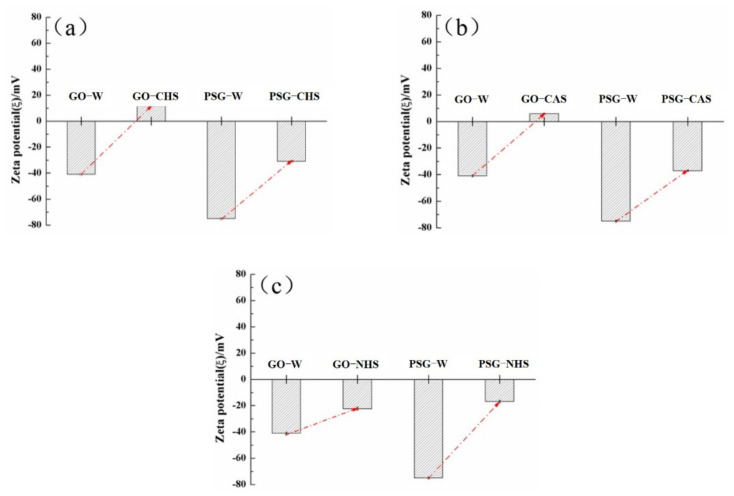
Average zeta potentials of GO and PSG particles in different solution systems: (**a**) CHS; (**b**) CAS; and (**c**) NHS.

It can be seen from Figure 13 that the zeta potentials of both the GO and PSG dispersed in distilled water were negative, indicating that the surfaces of GO and PSG particles were negatively charged. In addition, the zeta potential of PSG was significantly higher than that of GO, indicating that PSG was more stable than GO in the aqueous phase.

**Figure 14:**
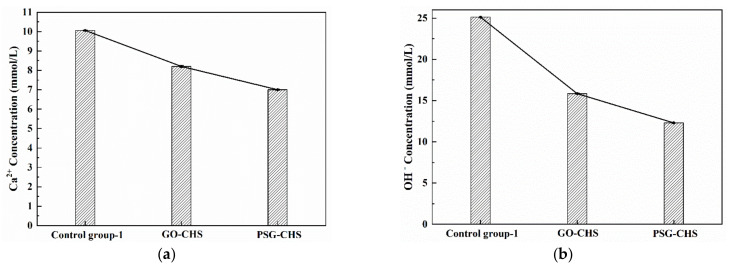
Ion concentrations of Ca(OH)_2_ solutions: (**a**) Ca^2+^ concentration; and (**b**) OH^−^ concentration.

Figure 14a shows that, after the GO was dispersed into the CHS solution, the zeta potential changed from a negative to a positive value with respect to GO-W, indicating that the surface electrical properties of the particles became positive. When the PSG dispersion was mixed into the CHS solution, the zeta potential decreased from −70 mV to −30 mV compared to PSG-W, showing that the particles still existed in a stable dispersion state [19,20]. The main cause of the above changes was the complexation of the -COOH groups on the surface of GO and PSG with Ca^2+^ in the system; this is discussed in Section 3.4.4.

The change in the zeta potential of GO and PSG in a Ca(NO_3_)_2_ solution (CAS) of equal Ca^2+^ concentration is shown in Figure 13b. The zeta potential of GO and PSG dispersed in the CAS solution was similar to that in Figure 13a, but the relative value changed less than that in the simulated pore (CHS, Ca(OH)_2_) solution. Compared with GO-W, GO-CAS had a positive zeta potential of approximately +15 mV, which was slightly less than the +25 mV of GO-CHS. In the PSG-CAS, the zeta potential decreased from −70 mV to −38 mV, and the amplitude was also slightly smaller than the zeta potential in PSG-CHS.

Figure 13c shows the zeta potential of GO and PSG in an NHS (NaOH) solution with equal OH^−^ concentration. Compared with GO-W and PSG-W, the zeta potentials of GO-NHS and PSG-NHS decreased significantly and, although both were negative, they were greater (more positive) than −25 mV. In addition, the zeta potential of PSG-NHS was slightly greater than that of GO-NHS.

#### 3.4.3. Effect of GO and PSG on the Ion Concentrations of Cement Solution

To better study the interaction between GO and PSG and ions in the system, the effects of varied ion concentrations in different systems were tested. Figure 14a,b shows the effect of GO and PSG on the concentration of Ca^2+^ and OH^−^ in a simulated pore (CHS) solution. As can be seen from Figure 14, the Ca^2+^ and OH^−^ concentration in GO-CHS and PSG-CHS decreased significantly compared to the control group, and these decreases were significantly greater than those in the GO-CHS group. These results showed that both GO and PSG can interact with the CHS solution by adsorbing significant concentrations of Ca^2+^ and OH^−^. Notably, when Ca^2+^ and OH^−^ were both present, PSG absorbed more Ca^2+^ and OH^−^ than GO.

**Figure 15:**
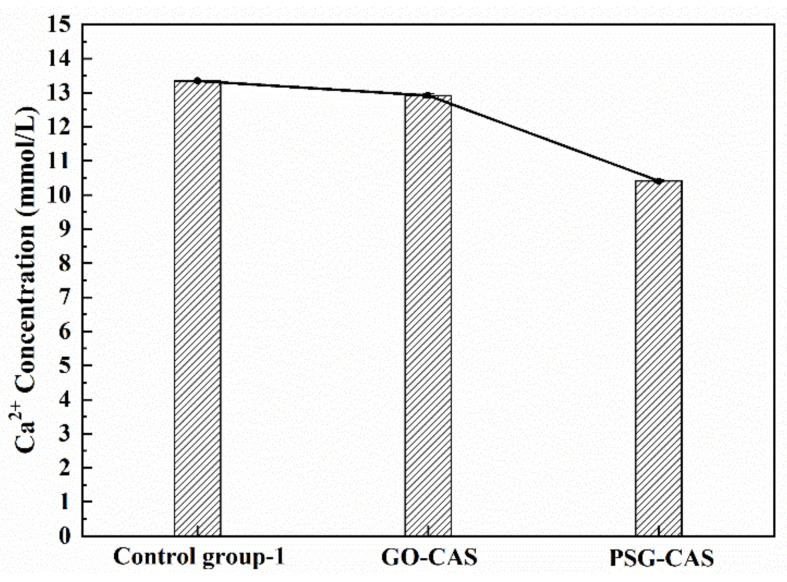
Ion concentrations in suspensions with Ca(NO_3_)_2_ solution with Ca^2+^ concentration equal to that in Ca(OH)_2_ solution(CHS).

To investigate whether there was a synergistic effect between Ca^2+^ and OH^−^, the ion concentrations in the two systems when each ion was present separately were also tested. Figure 15 shows the interaction of GO and PSG with Ca^2+^ in Ca(NO_3_)_2_ solution with a Ca^2+^ concentration equivalent to that in Ca(OH)_2_ (simulated pore) solution. It can be seen from Figure 15 that the Ca^2+^ concentrations in the GO-CAS and the PSG-CAS samples were both less than that in the control, which was similar to the results in the Ca(OH)_2_ solution shown in Figure 14. While this decrease of Ca^2+^ concentration in the two sample types was significantly lower than that in the Ca(OH)_2_ solution, the decrease in Ca^2+^ concentration in the GO-CAS sample was significantly less than that in the PSG-CAS sample.

**Figure 16:**
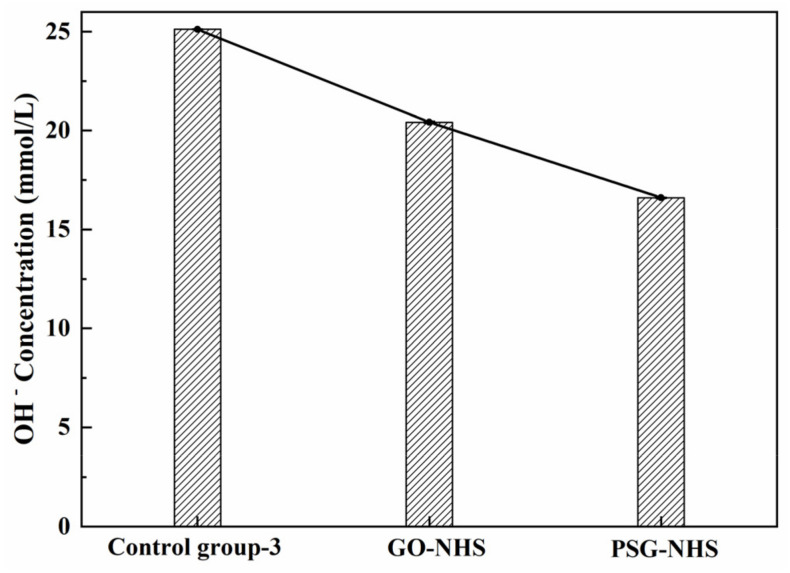
Ion concentrations in suspensions in sodium hydroxide solution (with OH^−^ concentrations equal to that in Ca(OH)_2_ (CHS) solution).

**Figure 17:**
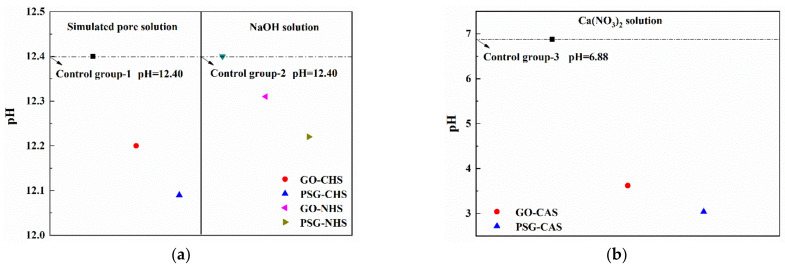
Effects of GO and PSG on pH in alkaline conditions and neutral conditions: (**a**) alkaline conditions; and (**b**) neutral conditions.

The effect of GO and PSG on the OH^−^ concentration in sodium hydroxide (NHS) solution (with an equal OH^−^ concentration to the CHS solution) is shown in Figure 17. It can be seen from Figure 16 that the OH^−^ concentration in the GO-NHS group and the PSG-NHS group were both less than in the control; this trend was similar to that in Figure 15, although the magnitude of the decrease was substantially less than that in the CHS solution. The results shown in Figure 15, Figure 16 and Figure 17 indicated that there may be a synergetic interaction between the complexation-driven adsorption of Ca^2+^ and OH^−^ on the surface of GO and PSG, related to the respective agglomeration properties of the GO and PSG sheets. The relationship between the complexation of Ca^2+^ by GO and PSG, the change in ion concentration, and the zeta potential is discussed in Section 3.4.4.

Figure 17 shows the pH changes of GO and PSG in these three systems. It can be seen from Figure 17 that the decrease of the pH value in the Ca(OH)_2_ solution with simultaneous presence of Ca^2+^ and OH^−^ was significantly higher than that in the NaOH solution with an equilibrium OH^−^ concentration, and that these results were consistent with those shown in Figure 14 and Figure 16.

#### 3.4.4. Holistic Analysis

The results in Figure 10 show that GO was prone to forming macroscopic aggregates in the simulated pore (Ca(OH)_2_) solutions and in the Ca(NO_3_)_2_ solutions with equivalent Ca^2+^ concentrations, and that the agglomerates formed in the Ca(OH)_2_ solution were larger and denser. In contrast, in the sodium hydroxide (NHS) solution with an equivalent OH^−^ concentration to the other solutions, GO did not form macroscopic agglomerates. The morphology of PSG did not change significantly in any of these systems. Thus, the presence of Ca^2+^ was a key factor in the agglomeration of GO in the cement paste, and OH^−^ synergized with Ca^2+^ to enhance agglomeration.

**Figure 18:**
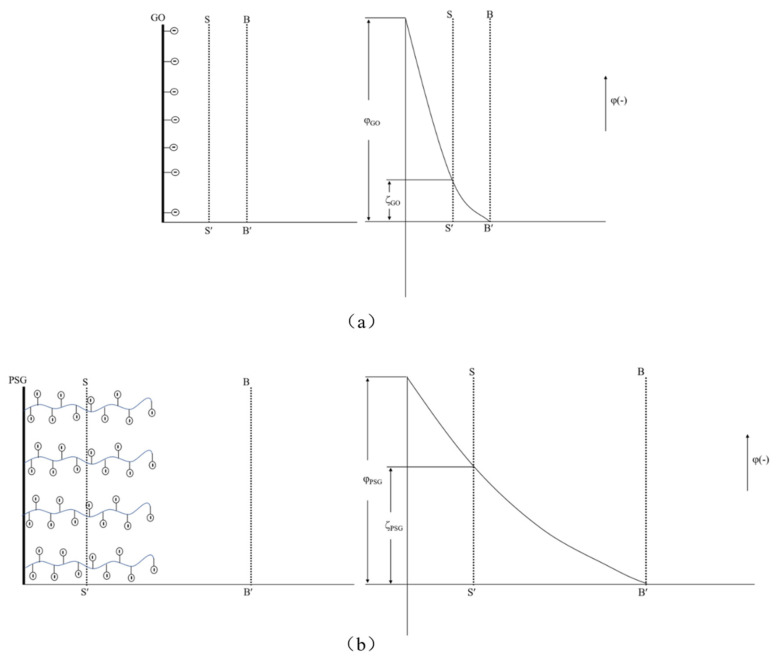
Zeta potential diagram of GO surface and PSG surface: (**a**) GO; and (**b**) PSG.

The zeta potential analysis results are shown in Figure 13. As can be seen, the aqueous dispersion of PSG was more stable than the aqueous dispersion of GO. This is in line with Gouy–Chapman electric double layer theory [21,22]. As shown in Figure 18a, the -COOH group contained on the surface of the GO was partially ionized in the aqueous phase system so that its surface was negatively charged, and thus the zeta potential at the sliding surface SS′ was ζ_GO_ (ζ_GO_ < 0). As shown in Figure 18b, the surface of PSG was modified with a certain proportion of polycarboxylate molecular chains; these chains contained a large number of -COOH groups, which, due to polymerization, generated more negative charge than GO. The carboxylic acid molecular chain extended outward so that the dense layer and the diffusion layer were epitaxial, and the rate of potential drop therefore decreased; thus, the zeta potential of the PSG at the sliding surface SS′ was ζ_PSG_ (ζ_PSG_ < 0). In the case of equal-sized sheets, |ζ_GO_| was less than |ζ_PSG_|, which is consistent with the actual situation in the analysis, as shown in Figure 14a.

When GO and PSG were doped into a system containing Ca^2+^, the COO^−^ groups contained on the surface of these materials complexed Ca^2+^ [23,24,25], thereby absorbing Ca^2+^ on the surface of the sheets. This complexation interaction affected the surface potential. The ion-concentration analysis results given in Section 3.4.4 also prove that GO and PSG complexed Ca^2+^.

**Figure 19:**
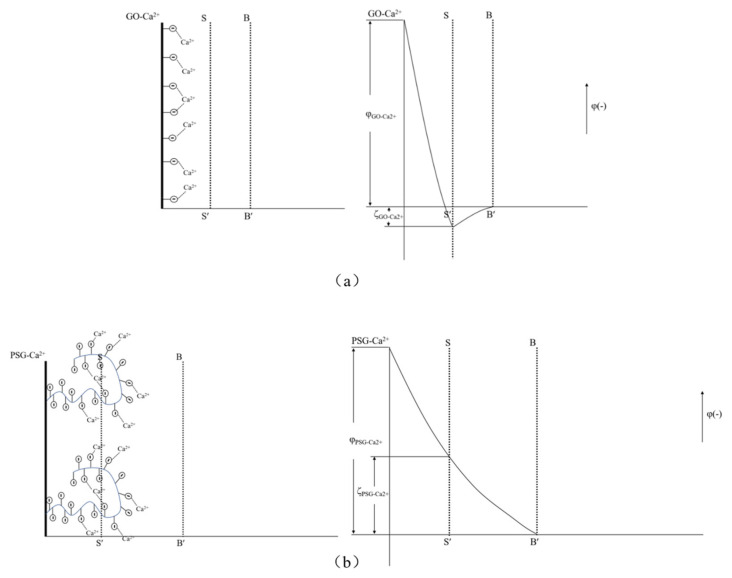
Zeta potential diagram of GO surface in Ca^2+^ system and PSG surface in Ca^2+^ ion system: (**a**) GO; and (**b**) PSG.

As shown in Figure 19a, Ca^2+^ was complexed and adsorbed on the COO- group of the GO surface. As there were only a few COO- groups on the GO surface, most were complexed with Ca^2+^, resulting in the potential of the GO surface becoming positive. However, due to the small size of Ca^2+^, the dense layer and the diffusion layer were not significantly epitaxial, that is, the positions of the SS surface and the BB surface were not substantially changed. Consequently, the positive potential caused by the complexation of Ca^2+^ caused the SS surface to gradually decrease until, like the BB surface, it was close to 0, and the potential at the BB surface was ζ_GO-Ca2+_ (ζ_GO-Ca2+_ > 0). These zeta potential results were consistent with those in Figure 14a,b. In addition, the fact that the potential of |ζ_GO-Ca2+_| was less than 25 mV showed that GO in this state could not stably exist in this dispersion system, which was consistent with its macroscopic agglomeration behavior.

In a PSG system containing Ca^2+^, as shown in Figure 19b, the -COO- group on the surface of the polycarboxylate molecular chain also complexed with Ca^2+^, increasing the rate of negative potential drop on the PSG surface. Under the action of Ca^2+^, a certain degree of involution of the molecular chain occurred, such that the dense layer and the diffusion layer moved back to the surface of the sheet [26]; this resulted in the potential at the SS surface being ζ_PSG-Ca2+_ (ζ_PSG-Ca2+_ < 0), and |ζ_PSG-Ca2+_| >25 mV, indicating that PSG was stable in this dispersion system.

When GO and PSG were incorporated into a system containing OH^−^, as shown in Figure 17a, OH^−^ deprotonated the -COOH group to -COO^−^, which exposed more negative charge. This phenomenon was illustrated by the negative potential shown in Figure 13c. Due to a certain amount of Na^+^ in the NaOH solutions, the negative potential of GO and PSG in this system still decreased somewhat. In addition, Na^+^ was more likely to be adsorbed onto a PSG sheet with more exposed COO^−^ groups, resulting in a greater potential drop in the PSG. Overall, the fact that the absolute values of both zeta potentials were greater than 25 mV showed that GO and PSG were stable in the NaOH solution.

**Figure 20:**
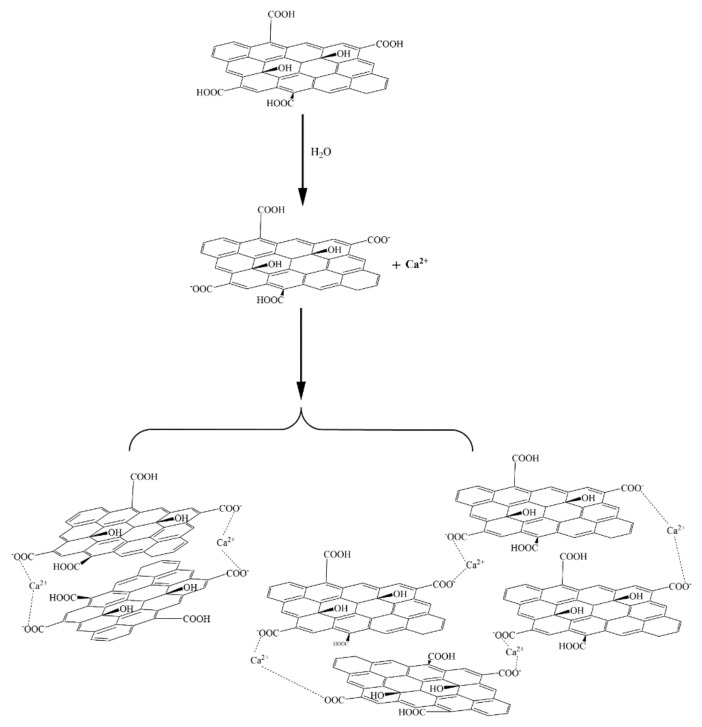
Formation of GO aggregates in Ca^2+^ solution.

The above results and analysis showed that the surface electrical properties of GO and PSG under the action of Ca^2+^ and OH^−^ were significantly different, and that this was directly related to the promotion of GO agglomeration and the inhibition of PSG agglomeration. As shown in Figure 20, the -COOH group on the GO surface was ionized to -COO^−^ in water, and as Ca^2+^ is a divalent metal ion, it complexed with two -COO^−^ groups, resulting in the agglomeration of positively charged GO sheets.

Zhao et al. [27] also suggested that the formation of GO agglomerates was related to the ability of Ca^2+^ to “bridge” GO sheets. Thus, it can be inferred that the black dot-like particles in the GO agglomerate in the ultra-depth microscope images (Figure 11) represent such Ca^2+^-bridging complexation. It can be seen that the number of these particles in the simulated pore ((Ca(OH)_2_; CHS) solution was significantly greater than in the Ca(NO_3_)_2_ solution with an equivalent concentration of Ca^2+^.

**Figure 21:**
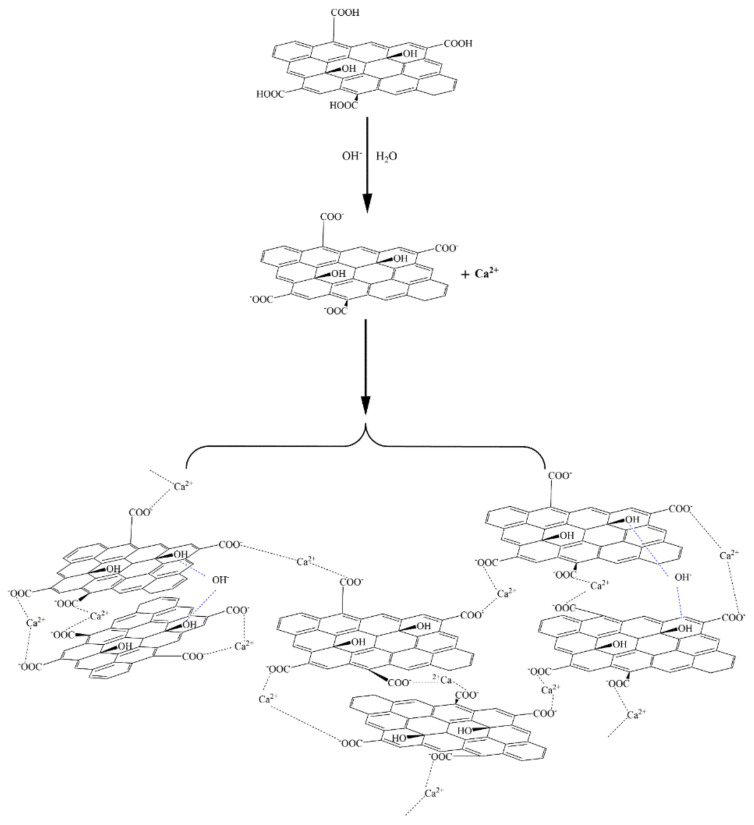
Formation of GO aggregates in simulated pore (Ca(OH)_2_) solution.

Due to the presence of both Ca^2+^ and OH^−^ in the Ca(OH)_2_ solution, some-COOH groups (which were otherwise not ionized on the GO surface) were deprotonated to COO-, as shown in Figure 21, which promoted the complexation of COO^−^ with Ca^2+^ and further increased the bridging effect of Ca^2+^. In addition, the OH^−^ formed a hydrogen bond with the -OH of the GO surface [28,29], further narrowing the distance between the sheets, such that the GO in the Ca(OH)_2_ solution formed a larger and denser agglomerate. However, the hydrogen bond did not play a dominant role due to the weakness of the hydrogen-bonding force, so the agglomeration phenomenon seen in Figure 11d was very weak. The OH^−^ also promoted the formation of hydrogen bonds between the -OH and water molecules on the GO surface, thereby synergistically affecting the fluidity and rheological properties of the cement slurry.

**Figure 22:**
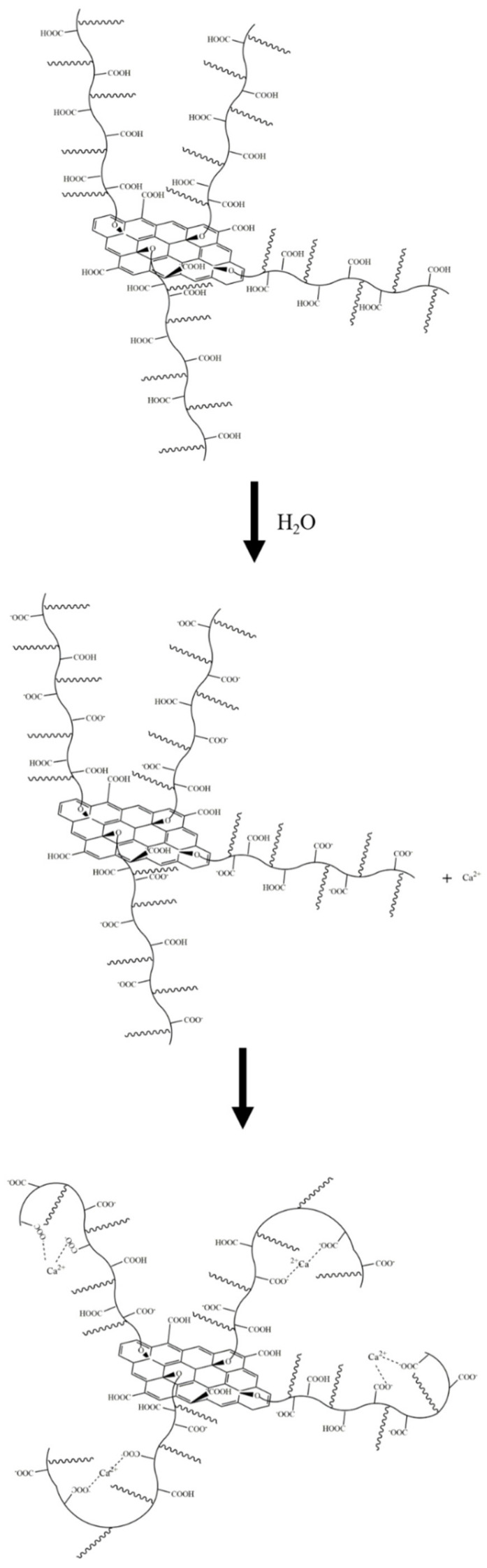
Mechanism of PSG complexation of Ca^2+^ in solution.

There was a larger number of -COO- groups on the polycarboxylate molecular chain on the PSG sheet than on the GO sheet, and these were available to complex with Ca^2+^, as shown in Figure 22. In this scenario, the binding of Ca^2+^ with the COO^−^ group of the long polycarboxylic chain of PSG caused the molecular chain to curl, thus destroying the Ca^2+^-bridge between the PSG layers. In addition, the carboxylic chains occupied the position of the OH on the GO surface, thus destroying the hydrogen bond between OH^−^ and water molecules. Together, these factors prevented the formation of agglomerates.

**Figure 23:**
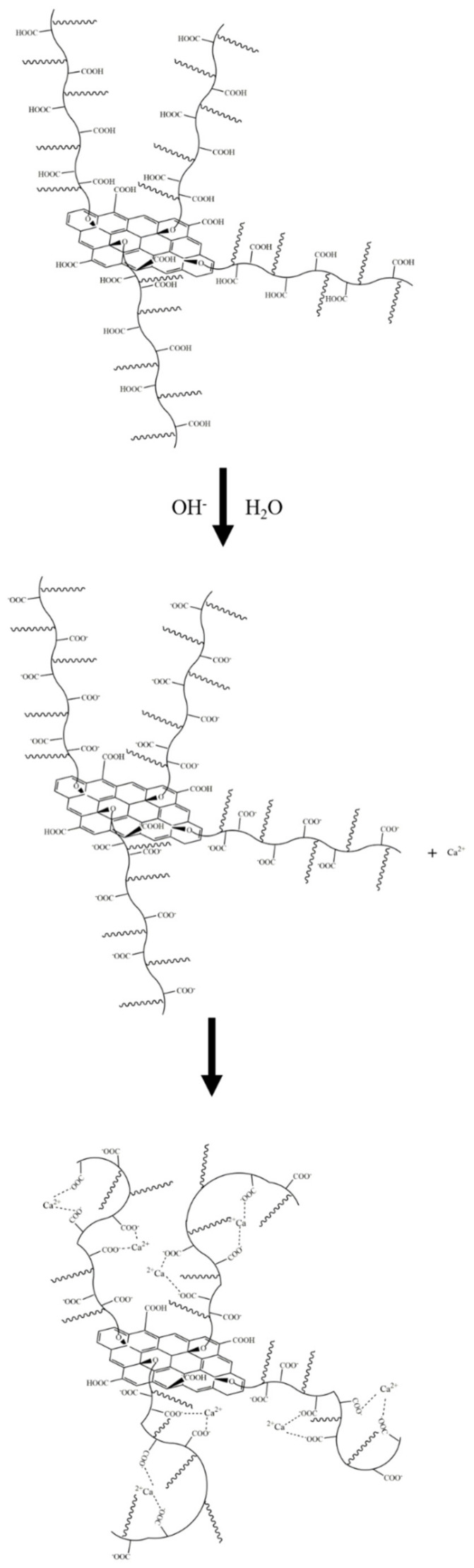
Diagram of action mechanism of PSG in Ca(OH)_2_ solution.

In the Ca(OH)_2_ solution in which Ca^2+^ ions and OH^−^ ions were both present, shown in Figure 23, the OH^−^ ion also captured the H^+^ of the -COOH group in the polycarboxylate molecules in the PSG, so that more COO^−^ groups were exposed and thus able to complex and adsorb more Ca^2+^. However, this Ca^2+^ was preferentially captured by the greater number of COO^−^ groups along the polycarboxylate molecular chains, thus preventing the bridging of layers by Ca^2+^, and inhibiting the formation of agglomerates.

**Figure 24:**
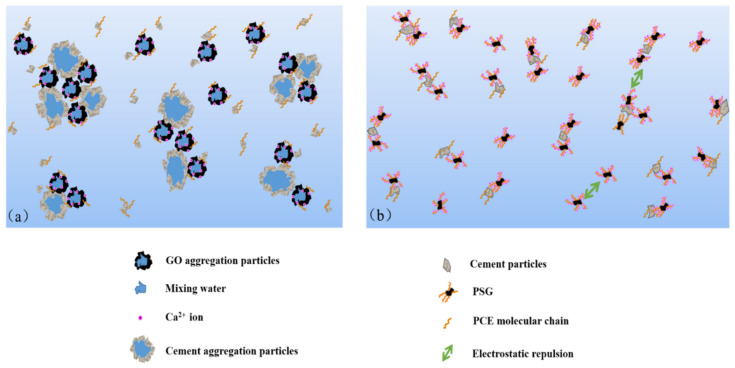
Schematic diagram of mechanism of GO and PSG on cement paste: (**a**) GO-cement system; and (**b**) PSG-cement system.

The above analysis shows that there were obvious differences in the behavior of GO and PSG in cement paste, consistent with their different effects on the slurry. As shown in Figure 24a, in the initial stage of cement-slurry mixing, GO rapidly reacted with Ca^2+^ and OH^−^ to form a large and dense agglomerate containing a certain amount of mixing water. Consequently, there was less free water in the slurry, and thus the fluidity of the slurry decreased. At the same time, the large specific surface area and positive surface charge of the agglomerates enabled them to adsorb some of the polycarboxylate superplasticizer, which resulted in an overall reduction in the superplasticizer. In addition, the agglomerates also interacted with cement particles via Ca^2+^ to form recombinant flocculation structures, thereby further affecting the fluidity and rheological parameters of the cement slurry.

When PSG was incorporated into a cement slurry, the internal complexation mentioned earlier, and the inhibition of agglomeration by the intramolecular chain, meant that agglomerates were not formed in the slurry aqueous phase, as shown in Figure 24b. The polycarboxylate molecular chains grafted on the surface of the PSG played a key role, by virtue of their electrostatic repulsion and steric hindrance, thereby preventing the adsorption of free polycarboxylate superplasticizer molecules on the surface of the sheet [30]. This enabled the superplasticizer to exert its normal dispersive effects, thus maintaining the fluidity of the slurry and generating rheological behavior comparable to that of the undoped slurry.

## 4. Conclusions

In this study, a PSG composite was successfully prepared by grafting polycarboxylate chains onto the surface of GO sheets. Meanwhile, both GO and PSG formed stable colloidal dispersions in aqueous dispersion systems. The influence of GO and PSG on cement pastes was investigated by fluidity analysis, rheological measurement, ion concentration analysis, and zeta potential measurement. This enabled the mechanisms through which GO and PSG act on cement paste to be determined. The main conclusions were as follows.

(1) With an increase in GO content, the initial fluidity of the cement paste decreased significantly, (with the maximum decrease being 42.2%), whereas the yield stress and plastic viscosity of the paste gradually increased (with the maximum respective increases being 152.80% and 126.89%, respectively).

In the aqueous phase of the cement paste, under the combined effect of the complexation of Ca^2+^ with -COO^−^ groups and the hydrogen bonds between OH^Å^ and hydroxyl groups, the GO sheets became linked, leading to large and dense agglomerates. The agglomerate structure, which strongly affected the fluidity and rheological properties of the cement pastes by sequestering a portion of the mixing water, adsorbed the superplasticizer molecules and formed a restructured flocculation structure with cement particles.

(2) The cement paste mixed with PSG did not show obvious thickening, which ensured the working performance of the paste and somewhat offset the negative effect of GO on the workability of the modified cement paste. After PSG was mixed into the cement slurry, the polycarboxylate molecular chains grafted onto its surface complexed with Ca^2+^ and coiled inward, breaking the bridging effect between the sheets and preventing the formation of aggregates, thus ensuring efficient PSG dispersal in the cement paste. Moreover, the electrostatic repulsive force between the polycarboxylate molecular chains on the surface of PSG and the superplasticizer molecules in the paste prevented the superplasticizer molecules from adsorbing onto the PSG sheets, and thus the superplasticizer acted solely on the cement particles, guaranteeing the working performance of the cement paste.
(3) PSG effectively decreased the thickening effect of GO on cement paste, providing a new route for the application of graphene materials in the field of cement-based materials.
(4) This study has achieved the expected aim, but there are still limitations due to the single type of coupling components and the molecular structure of polycarboxylate. Based on this study, the influence of types of coupling agents and the molecular structure of polycarboxylate should be widely investigated in future studies, and some functional components should be introduced to enhance the function properties of modified GO.

## Figures and Tables

**Table 1:** Chemical and mineral composition of reference cement clinker (%).

SiO_2_	Al_2_O_3_	Fe_2_O_3_	CaO	MgO	SO_3_	NaO-eq	f-CaO	C_3_S	C_2_S	C_3_A	C_4_AF
22.49	4.57	3.38	65.52	2.31	0.35	0.51	0.76	56.23	22.08	6.40	10.28

**Table 2:** Main physicochemical parameters of TPEG.

Appearance	Hydroxyl Value (mg/g)	Unsaturation (mol/mg)	pH Value (1% Aqueous Solution)	Relative Molecular Mass (g/mol)
Light-yellow flake	23.4–26.7	0.37	5.0–7.0	2400

**Table 3:** Composition of cement-compound pastes.

Group	Cement (g)	Water (g)	GO Dispersions (g)	PSG Dispersions (g)	PCE (g)
Blank	300	88.5	-	-	
GO-0.01		73.5	15	-	
GO-0.03		43.5	45	-	1.8
PSG-0.01		73.5	-	15	
PSG-0.03		43.5	-	45	

**Table 4:** Composition of Ion-Concentration Test Samples.

	aq. Ca(OH)_2_ (g)	aq. Ca(NO_3_)_2_ (g)	aq. NaOH (g)	GO (g)	PSG (g)	Deionized Water (g)
Control group-1	10	-	-	-	-	10
Control group-2	-	10	-	-	-	10
Control group-3	-	-	10	-	-	10
GO-W	-	-	-	10	-	10
PSG-W	-	-	-	-	10	10
GO-CHS	10	-	-	10	-	-
PSG-CHS	10	-	-	-	10	-
GO-CAS	-	10	-	10	-	-
PSG-CAS	-	10	-	-	10	-
GO-NHS	-	-	10	10	-	-
PSG-NHS	-	-	10	-	10	-

Note: The Ca(NO_3_)_2_ solutions (CAS group) and NaOH solutions (NHS group) had the same Ca^2+^ and OH^-^ concentrations as the Ca(OH)_2_ solutions (CHS group), as the primary purpose of this analysis was to investigate the separate effects of Ca^2+^ and OH^−^ on the specimens. GO-W/CHS/CAS/NHS: it means to mix GO into Deionized water/Ca(OH)_2_ solutions/Ca(NO_3_)_2_ solutions/NaOH solutions. PSG-W/CHS/CAS/NHS: it means to mix PSG into Deionized water/Ca(OH)_2_ solutions/Ca(NO_3_)_2_ solutions/NaOH solutions.

**Table 5:** Fitting result of M–B Model parameters.

Group	Fitting Equation	*R* ^2^	τ_0_/Pa	SDτ_0_	η_p_/Pa·s	SDη_p_
Blank	τ = 11.012 + 0.331γ + 4.743 × 10^−4^γ^2^	0.998	11.012	0.984	0.331	0.018
GO-0.01	τ = 14.224 + 0.444γ + 4.476 × 10^−4^γ^2^	0.998	14.224	0.862	0.444	0.016
GO-0.03	τ = 27.838 + 0.751γ + 1.558 × 10^−4^γ^2^	0.997	27.838	1.196	0.751	0.039
PSG-0.01	τ = 12.157 + 0.303γ + 7.319 × 10^−4^γ^2^	0.998	12.157	1.128	0.303	0.023
PSG-0.03	τ = 14.412 + 0.352γ + 5.971 × 10^−4^γ^2^	0.998	14.412	1.091	0.352	0.019

**Table 6:** Fitting results of H–B model parameters.

Group	Fitting Equation	*R* ^2^	τ_0_/Pa	SDτ_0_	k	SDk	n	SDn
Blank	τ = 12.337 + 0.143γ^1.204^	0.997	12.337	1.454	0.143	0.036	1.204	0.045
GO-0.01	τ = 15.460 + 0.038γ^1.149^	0.996	15.460	1.210	0.038	0.038	1.149	0.028
GO-0.03	τ = 28.833 + 0.723γ^1.008^	0.997	28.833	2.756	0.723	0.157	1.008	0.038
PSG-0.01	τ = 11.243 + 0.080γ^1.222^	0.995	11.243	1.727	0.080	0.024	1.222	0.054
PSG-0.03	τ = 14.155 + 0.130γ^1.141^	0.997	14.155	1.526	0.130	0.031	1.141	0.042
